# Immunosuppression and Opportunistic Infections: A Rare Case Report of Nocardia Osteomyelitis of the Pelvis

**DOI:** 10.7759/cureus.45306

**Published:** 2023-09-15

**Authors:** Richard I Suarez, Michaela Polmann, Lorena Del Pilar Bonilla, Carlos G Torres-Viera, Kebir Bedran

**Affiliations:** 1 Health Policy, Herbert Wertheim College of Medicine, Florida International University, Miami, USA; 2 Medicine, Herbert Wertheim College of Medicine, Florida International University, Miami, USA; 3 Hospital Medicine, Baptist Health South Florida, Miami, USA; 4 Infectious Disease, Baptist Health South Florida, Miami, USA

**Keywords:** infection spread, atypical infection, immuno suppresion, nocardia species, osteo-myelitis

## Abstract

Patients with a long-standing history of immunosuppression are at significantly increased risk of opportunistic infections. One such group of organisms that may cause these types of infections includes the *Nocardia* genus, a gram-positive, filamentous rod that demonstrates a branching pattern, is urease-producing and has acid-fast properties. The disease profile of *Nocardia* varies with manifestations ranging from cutaneous infection to severe pulmonary or central nervous system (CNS) infections, and rarely, osteomyelitis. In this case report, we present an 87-year-old female with persistent left gluteal and lumbar pain, generalized body aches, chills, and fevers diagnosed with *Nocardia asiatica* osteomyelitis of the pelvis, likely secondary to dissemination from pulmonary cavitary disease in an immunosuppressed host with chronic neutropenia. On magnetic resonance imaging (MRI), the patient was found to have heterogeneous enhancement, central necrosis, and loss of cortical margins of the left iliac wing, alongside a rim-enhancing soft tissue mass from the left iliac bone into the left gluteal soft tissues and left paraspinal musculature representing an abscess. She was promptly treated with surgical irrigation and drainage with surgical wound cultures growing *Nocardia asiatica*. She received treatment with trimethoprim-sulfamethoxazole antibiotics with symptom improvement and is following up with an infectious disease physician outpatient. Management of osteomyelitis, like in this case, involves long-term antibiotics with the potential need for surgical intervention. There are few reported cases of extrapulmonary *Nocardia* infections, particularly osteomyelitis, demonstrating the importance of their inclusion in the literature to better serve patients to allow for timely intervention for rare and life-threatening conditions. In immunocompromised hosts, the differential diagnosis should include opportunistic infections and less common pathogens, especially in those with atypical presentations, including gluteal and leg pain.

## Introduction

Patients with a long-standing history of immunosuppression, secondary to a medical condition or medication-induced, are at significantly increased risk of opportunistic infections. These types of infections are normally diminished by an intact immune system, but when these defense mechanisms are reduced, these pathogens can cause infection. One such group of organisms that may cause opportunistic infections includes the *Nocardia* genus. Over 100 species of *Nocardia* exist with about half being known to cause infections in humans or animals [1]. This bacterium is a Gram-positive, filamentous rod that demonstrates a branching pattern, is urease-producing, and has acid-fast properties [2,3]. It is normally found to be ubiquitous in the environment, specifically within the soil, and is usually spread by inhalation of this environmental debris [1,2]. Though this organism most often infects immunocompromised hosts, it is predicted that up to one-third of all reported cases are in immunocompetent individuals [2].

The disease profile of *Nocardia* varies with manifestations ranging from pulmonary infections - which is the predominant form of the disease (62% to 80% of cases) - to cutaneous infection - often secondary to traumatic inoculation - or central nervous system (CNS) infections, and rarely, osteomyelitis [1-4]. When the infection does involve the bone, it is often due to bacteremia or trauma. In the literature, it has been reported in the spine, sternum, femur, tibia, femorotibial interface, sacrum, and skull [5-10]. Osteomyelitis of the hip caused by a *Nocardia* species is very rare and has been reported only once recently in an elderly woman without known immunosuppression or characteristic respiratory or CNS infections resulting in dissemination to the pubis [11]. Overall, there are few reported cases of extrapulmonary *Nocardia* infection, demonstrating the importance of their inclusion in the literature to better serve patients to allow for timely intervention for potentially rare and life-threatening conditions. In this article, we present an 87-year-old female with *Nocardia* osteomyelitis of the pelvis, likely secondary to dissemination from classic pulmonary cavitary disease in an immunosuppressed host.

## Case presentation

**Figure 1:**
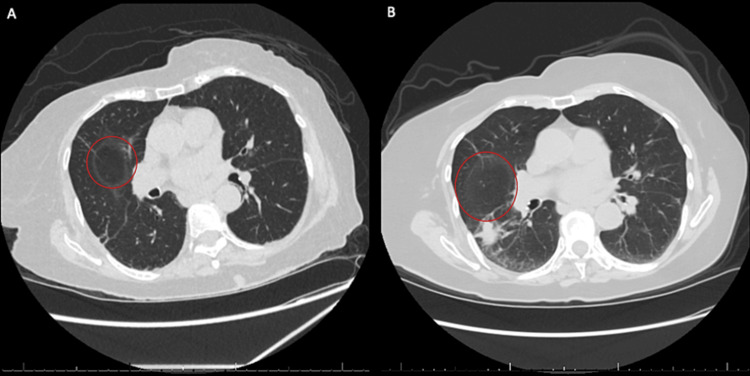
CT chest without contrast demonstrating pulmonary findings of the right lower lobe Cavitary lesion denoted with red circles. (A) CT from current admission showing interval development of cavitary lesion. (B) CT chest from previous admission one week prior with right lower lobe consolidation.

The patient is an 87-year-old female with a significant past medical history of severe aortic stenosis, hypertension, hyperlipidemia, first-degree AV block, chronic neutropenia (unclear etiology, but possible T-cell lymphoproliferative disorder with T-cell receptor gamma gene rearrangement positive mutation and low CD4/CD8 ratio), anemia, and history of disseminated cryptococcus with fungemia and meningitis in 2016. In the emergency department, the patient was evaluated for cough, generalized body aches, chills, fevers, nausea, and left lumbar/gluteal pain radiating to the posterior aspect of the left lower extremity. She denied recent travel history, exposure to known sick contacts, or significant past surgical history. The patient was admitted to the hospital one week before due to a right lower lobe pneumonia and was discharged on cefdinir but returned since symptoms were not improving. On initial examination, she was well appearing with an aortic systolic ejection murmur and significant pain to palpation in the left sacral area. Vital signs demonstrated temperature of 38.1 °C, heart rate of 118, respiratory rate of 18, blood pressure of 110/59mmHg, and oxygen saturation on room air of 96%. Table 1 summarizes the laboratory tests performed. All other laboratory values were unremarkable, including liver function tests. The computed tomography (CT) scan of the chest without contrast revealed interval development of central cavitation in the right lower lobe consolidation as compared to a previous CT chest one week prior (Figures 1A, 1B).

**Table 1:** Patient laboratory results Significant values are bolded. Hgb: Hemoglobin, HCT: Hematocrit, WBC: White blood cell, Na: Sodium, Cr: Creatinine, CrCl: Creatinine clearance, BUN: Blood urea nitrogen, PCT: Procalcitonin, CRP: C-Reactive protein, ESR: Erythrocyte sedimentation rate.

Laboratory Tests	Result
Hgb	12.2 g/dL
Hct	38.3%
WBC	2.19 K/µL (reduced)
Platelet	402 K/µL (elevated)
Na	133 mmol/L (reduced)
Cr	0.7 mg/dL
CrCl	38.06 mL/min (reduced)
BUN	14 mg/dL
PCT	0.15 ng/mL (elevated)
Lactic Acid	2 mmol/L
CRP	243 mg/dL (elevated)
ESR	64 mm/hr (elevated)

The patient was admitted for persistent right lower lobe pneumonia with the development of central cavitation. She was started on cefepime for broad-spectrum antibiotic coverage. The respiratory pathogens panel, including viral, bacterial, and fungal organisms, was completed. The viral panel was negative. *Streptococcus pneumoniae* and *Legionella* species urinary antigens were both negative. Methicillin-resistant *Staphylococcus aureus* (MRSA) was not detected on the nasal screen. *Aspergillus galactomannan* and Beta-d-glucan tests were negative. *Cryptococcus* antigen was positive with a titer 1:8; however, this was decreased compared to the previous result, suggesting that this presentation was less likely to be due to cryptococcal disease. Blood cultures were negative; however, the patient had several days of antibiotic treatment in the outpatient setting before admission, which may have resulted in a false negative result. There were initial concerns for the recurrence of cryptococcal illness, endocarditis, and unusual microorganisms in a patient with chronic neutropenia, which prompted an infectious diseases consult.

**Figure 2:**
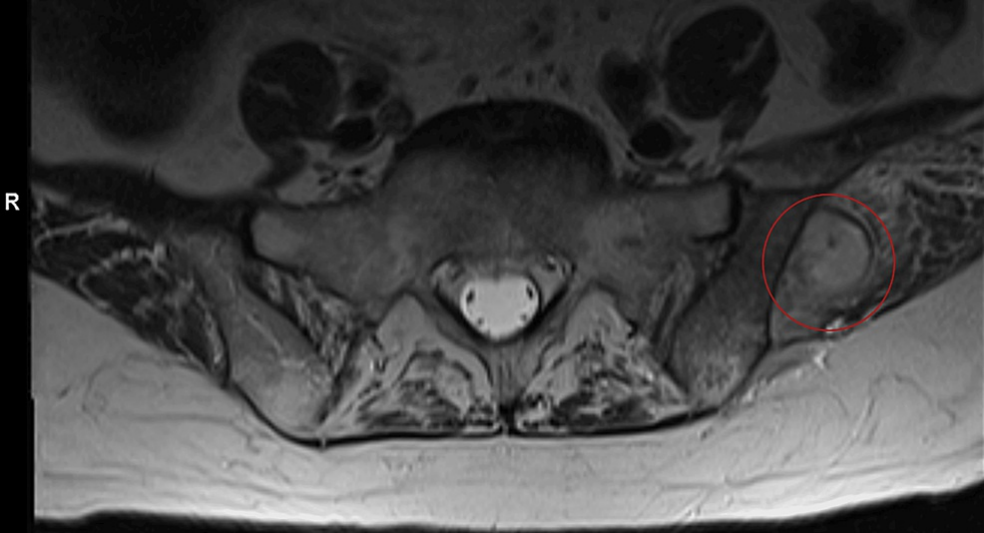
Lumbar MRI demonstrating osteomyelitis Potential abscess denoted with a red circle. The radiology report described acute osteomyelitis of the left iliac bone with multiple hypodensities surrounded by enhancement which may involve the left posterior paraspinal musculature at the level of L5. Suspicion for infectious process, possible abscess with probable sacroiliac involvement, and a lobulated gluteal abscess.

**Figure 3:**
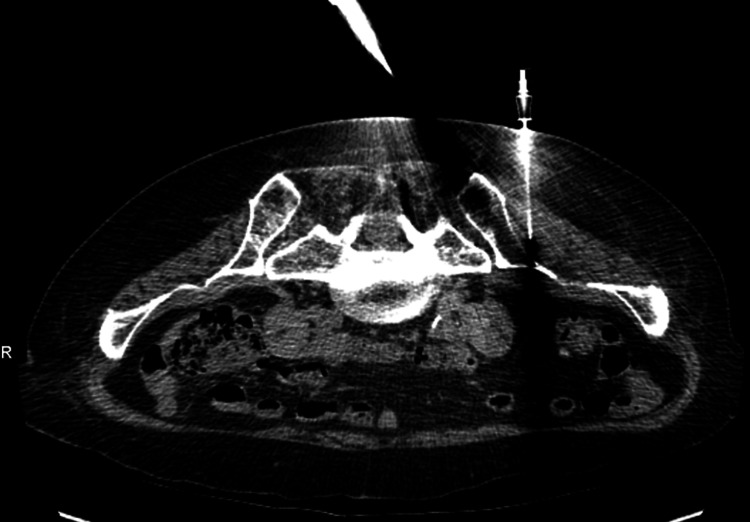
CT-guided aspiration of left iliac bone/left gluteal abscess Needle identifying site of sample extraction.

Due to persistent left lumbar and gluteal pain, MRI with and without contrast of the lumbar spine was performed, revealing a possible abscess or other infectious process involving the left iliac bone and adjacent muscle (Figure 2). As a result, she was started on daptomycin for suspected acute osteomyelitis of the left iliac bone and possible involvement of the left posterior paraspinal musculature at the level of L5 and a lobulated gluteal abscess. The patient subsequently underwent successful CT-guided aspiration and fine-needle biopsy of the left gluteal abscess with samples of bone and soft tissue (Figure 3). Bone and soft tissue samples demonstrated acute inflammation consistent with acute osteomyelitis.

**Figure 4:**
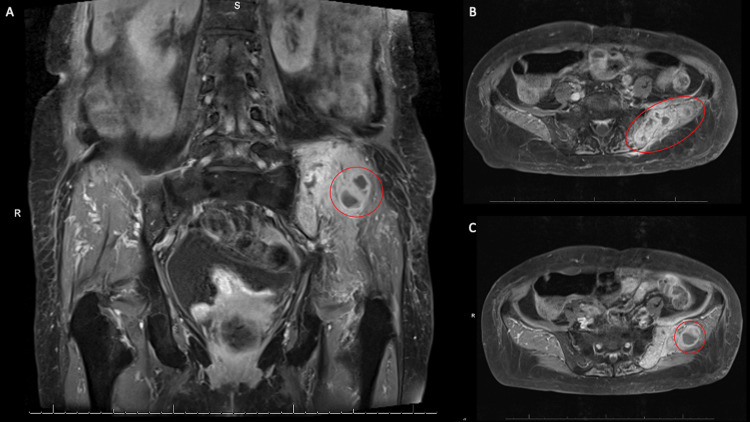
Pelvic MRI demonstrating osteomyelitis Red circles denote areas of interest. (A) The coronal section showing the infectious process on the left. (B) Axial section showing osteomyelitis of the left iliac bone. (C) Axial section showing left gluteal abscess. Radiology report described diffuse marrow replacement of the left posterior superior iliac spine extending into the left iliac wing with heterogeneous enhancement, central necrosis, and loss of cortical margins. This process appears to cross the sacroiliac joint with focal involvement of the subchondral left sacral ala and is favored to represent an osteomyelitis. Rim-enhancing soft tissue decompresses from the left iliac bone into the left gluteal soft tissues, left paraspinal musculature and subcutaneous soft tissues is favored to represent abscess formation. Mild edema and enhancement along the undersurface of the left iliacus muscle belly.

In order to further evaluate the area of interest, a pelvic MRI with contrast was completed, which further supported the diagnosis (Figures 4A-4C). The patient was taken to the operating room by the orthopedic surgery team for left ilium irrigation and debridement. Surgical fluid Gram stain revealed few white blood cells, no epithelial cells, and no organisms. The culture demonstrated light growth of a gram-positive rod with a positive modified acid-fast smear. The bacteria were ultimately identified as *Nocardia asiatica*. Considering these findings, the patient’s persistent pneumonia with central cavitation despite previous antibiotic treatment, and history of immunosuppression due to chronic neutropenia, we suspect her pelvic *Nocardia* osteomyelitis was a result of hematogenous spread from the lung. She was started on intravenous trimethoprim-sulfamethoxazole 5 mg/kg/day with the plan to complete at least six weeks of this antibiotic therapy. She is following up with an infectious disease's physician outpatient.

## Discussion

Osteomyelitis is an infection of bone, typically caused by a direct inoculation from trauma, by contiguous spread from nearby infected tissue, or by hematogenous spread due to bacteremia [12]. Individuals with diabetes mellitus may be at increased risk for developing osteomyelitis due to decreased blood flow to the extremities, impaired wound healing, and the presence of peripheral neuropathy that may cause trauma in the extremities to initially go unnoticed [12]. In this case, we suspect that our patient’s osteomyelitis was a result of hematogenous spread from a pulmonary infection. Although blood cultures were negative, the isolation of *Nocardia* from routine blood cultures, despite its propensity for dissemination, is rare. Moreover, the patient had received extensive outpatient antibiotic treatment which may have affected these results.

Acute osteomyelitis typically presents with dull pain at the site of infection and may be accompanied by fever. If the source of osteomyelitis is trauma or contiguous spread, the site of infection will often be erythematous, tender, and warm to the touch. The patient in this case presented with signs of fever and dull pain in the left lumbar and gluteal area worsened with ambulation or standing. She experienced severe pain upon direct palpation of the area, but there were no skin traumatic or inflammatory changes on inspection. Chronic osteomyelitis presents with similar symptoms, although symptom duration is often longer and more insidious, and constitutional symptoms like fever are less common [12]. The most common causative pathogen of osteomyelitis is *S. aureus*, with an increasing prevalence of MRSA. Coagulase-negative staphylococci, beta-hemolytic streptococci, enterococci, and gram-negative bacilli are also common causes of osteomyelitis [12]. Immunocompromised patients, such as the patient presented in this case, are at increased risk of infection from less common pathogens. Immunocompromised patients may develop osteomyelitis from tuberculous and non-tuberculous mycobacteria and fungi such as *Candida*, *Blastomyces*, *Coccidioides*, *Cryptococcus*, and *Aspergillus* [12]. *Nocardia* is also a causative organism, as demonstrated by this case report, but is much rarer than the aforementioned pathogens. 

The pelvic bone is an unusual location for osteomyelitis and therefore may result in delayed diagnosis of the condition. Pelvic osteomyelitis should be considered if a patient has signs of inflammation such as fever elevated acute phase reactants in the blood, and pelvic pain that worsens with walking or other exercise. The pain may radiate to the suprapubic, perineal, or inguinal area [13]. It may even present as abdominal pain with guarding, mimicking acute appendicitis [13]. 

Treatment of osteomyelitis involves surgical debridement of the infected bone and treatment with parenteral antibiotics for at least four to six weeks [12]. However, if a significant amount of infected bone remains after surgery, a more prolonged course of antibiotics will likely be necessary. In this case, our patient underwent extensive debridement of the necrotic portions of the left iliac bone for source control of the infection. She will be treated with at least six weeks of antibiotics for her osteomyelitis. 

## Conclusions

Unusual organisms should be considered causative agents for infection in immunocompromised patients. Lack of improvement in pneumonia symptoms or development of new symptoms despite treatment should prompt concern for a more complicated infection. A patient with pneumonia presenting with new onset localized pain should receive imaging to evaluate for osteomyelitis. A favorable prognosis and shorter duration of treatment are heavily dependent on early identification of osteomyelitis.
